# High-Energy, Short-Duration Bursts of Coherent Terahertz Radiation from an Embedded Plasma Dipole

**DOI:** 10.1038/s41598-017-18399-3

**Published:** 2018-01-09

**Authors:** Kyu Been Kwon, Teyoun Kang, Hyung Seon Song, Young-Kuk Kim, Bernhard Ersfeld, Dino A. Jaroszynski, Min Sup Hur

**Affiliations:** 1 School of Natural Science, UNIST, 50 UNIST-gil, Ulju-gun, Ulsan, 44919 Korea; 2 Department of Physics, Scottish Universities Physics Alliance and University of Strathclyde, Glasgow, G4 0NG UK

## Abstract

Emission of radiation from electrons undergoing plasma oscillations (POs) at the plasma frequency has attracted interest because of the existence of intriguing and non-trivial coupling mechanism between the electrostatic PO and the emitted electromagnetic wave. While broadband emission from plasma waves in inhomogeneous plasma is well known, the underlying physics of narrowband emission at the plasma frequency observed in experiments and in solar radio-bursts is obscure. Here we show that a spatially-localized plasma dipole oscillation (PDO) can be generated when electrons are trapped in a moving train of potential wells produced by the ponderomotive force of two slightly detuned laser pulses that collide in plasma and give rise to a burst of quasi-monochromatic radiation. The energy radiated in the terahertz spectral region can reach an unprecedented several millijoules, which makes it suitable for applications requiring short pulses of high-intensity, narrowband terahertz radiation.

## Introduction

The quest for powerful radiation sources is driven by the need for time-resolved tools that give access to nonlinear behaviour, which enables probing of the evolution of the structure of matter subject to stimuli. Several types of radiation sources are available in the terahertz spectral range. The most powerful of these includes free-electron lasers (FELs), which are intrinsically tuneable sources based on the interaction between relativistic electron beams and undulators; but they are very expensive and large devices. More widely available and compact radiation sources are usually based on solid-state media, but they only produce relatively low energies because of limitations to the maximum internal electric fields that they can withstand. However, plasma, where charged particles have intrinsic oscillatory properties and can oscillate locally at the plasma frequency if perturbed, can withstand extremely high fields. It is the basis for many technologies, but still poses many puzzles, such as in astrophysics where there are open questions on the origins of the large amounts of energy observed in cosmic radio-bursts.

The excitation of a localised oscillation of electrons in plasma, a plasma dipole, would have almost ideal characteristics as a radiation source, if it could be excited and its energy extracted from the plasma. Its frequency would also be narrowband and equal to the plasma frequency (${\omega }_{p}\propto \sqrt{n}$), which is readily tuned from gigahertz to terahertz frequencies, simply by changing the plasma density *n*. Internal electric fields in plasma can exceed GV/cm without material damage, which gives the potential of radiating very large fields. However, there are still outstanding questions on whether an electromagnetic wave can be emitted by an embedded plasma oscillation (PO), in the first place. These questions are non-trivial, because the plasma oscillator, in contrast to ubiquitous dipole radiators in vacuum (e.g. dipole antenna), is surrounded by plasma, which generally cuts-off propagation of electromagnetic waves with frequencies at or below the plasma frequency. Raman scattering in plasma is a good example of radiation arising from driven electrons in plasma^1^, but this is always well above the cut-off frequency. However, despite repeated observations of radiation at the plasma frequency in experiments and simulations^2–7^, its interpretation remains unclear^8–10^. It has been shown that a plasma oscillation can emit radiation in inhomogeneous plasma, via, for example, inverse mode conversion^11–15^. It is clear that there is a significant interest in the mechanisms of radiation. In the astrophysical literature, radiation at the local plasma frequency in inhomogeneous plasma (and the accompanying frequency drift due to density variations and plasma dispersion) is generally accepted as one of the origins of solar radio-bursts (e.g. type III) from the coronal plasma^16–21^.

Interpreting how radiation is emitted from oscillating electrons in plasma can lead to confusion and usually requires detailed information on the radiating system and its environment. For example, if a PO is considered as a travelling *wave*, it can radiate through mode-conversion at suitably shaped plasma boundaries, but this is inevitably broadband^13,14^, which contrasts with emission from a *spatially-localized oscillation* of a block of electrons. As long as the dimension of the dipole is small compared with the wavelength (2*πc*/*ω*_*p*_), the plasma dipole oscillation (PDO) is similar to the oscillation of a point charge, which will emit radiation if it is located sufficiently close to the plasma-vacuum boundary.

**Figure 1:**
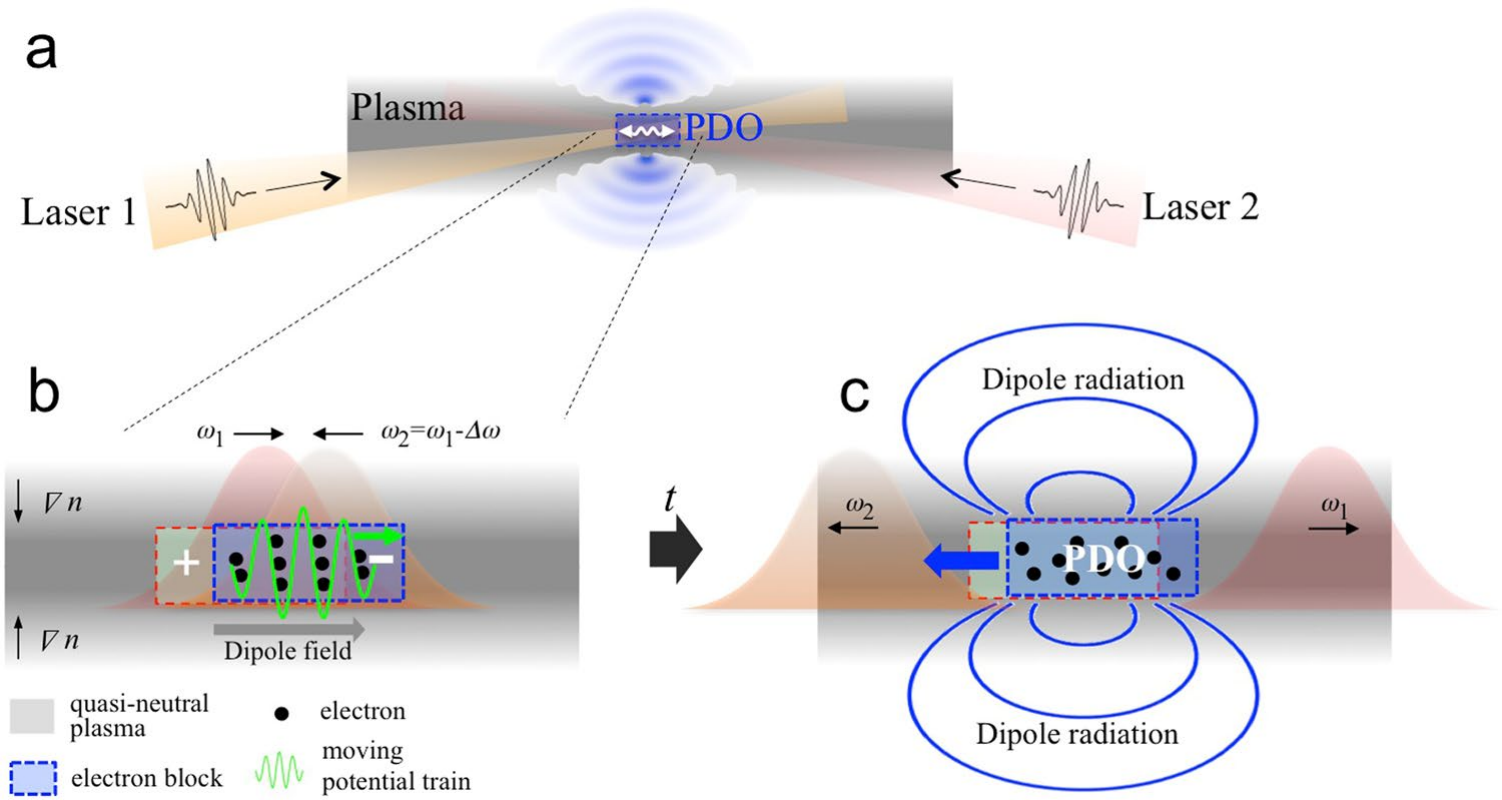
Generation of the PDO and its emitted radiation. (**a**). A dipole moment is built up in the overlap region of the colliding laser pulses. (**b)** Generation of a dipole moment in plasma by the electrons trapped in a moving potential train. (**c**). After the laser pulses cross each other, the dipole block continues to oscillate. Radiation is emitted transversely through a narrow layer of plasma during the dipole build-up period and successive plasma oscillations.

Although the concept of the PDO is commonly employed in pedagogical descriptions of POs (e.g. the slab model in ref.^22^), the existence of a long-term persisting, strong-field PDO in practical systems has not been previously proposed. We demonstrate, for the first time, that it is indeed possible to generate a very strong PDO that emits a quasi-narrowband burst of radiation with high efficiency, and with an adjustable frequency up to ≈20 terahertz (THz) and energies of several millijoules, derived from joule-level pump energy. The proposed mechanism for generating a PDO is the build-up of a dipole moment by trapping electrons in a spatially localized, moving train of potential wells. The trapped electrons ride the potential wave and are *displaced*, which generates a dipole field through charge separation between the displaced electron-block and the remaining and quasi-stationary ions (Fig. 1). As a novel method of producing the moving potential train, we consider the ponderomotive (PM) potential associated with the beat wave of two slightly detuned, ultrashort, laser pulses colliding in plasma (Fig. 1). In the region where the ultrashort pulses overlap, electrons are trapped by the PM potential wells that move at a phase velocity *v*_*ϕ*_ = (*ω*_1_ − *ω*_2_)/(*k*_1_ + *k*_2_), where *ω*_1,2_ and *k*_1,2_ are the angular frequencies and wavenumbers of the laser pulses, respectively. Later, after the laser pulses have crossed each other, the moving potential diminishes, and the displaced electron-block is *released* to commence a weakly damped plasma dipole oscillation. The axial and transverse dimensions of the dipole are comparable, respectively, to the longitudinal dimension and the transverse spot size of the driving pulses. Therefore, the dipole can be made point-like (*i.e*. compared with the radiation wavelength) by choosing ultrashort, sharply focused driving pulses.

When the point-like plasma dipole is located very close to the plasma-vacuum boundary, shielding of the radiation by the thin plasma layer between the dipole and vacuum is negligible. In this case, the PDO emits radiation similar to that of a regular dipole in (semi-infinite) vacuum.

While broadband, weakly-tuneable, sub-millijoule bursts can currently be generated from laser-solid interactions^14,23,24^, it has not been possible to concentrate millijoule energies into a relatively narrowband spectrum with high tuneability. The unprecedented high spectral density concentrated at a few to tens of terahertz makes this suitable for applications requiring strong terahertz fields^25–28^. FELs can be tuned, but usually have a macropulse structure and have limited micropulse energy per micro-pulse, and in addition are large and expensive. A compact narrowband source of THz radiation is the quantum cascade laser (QCL) that can provide continuous-wave (cw), monochromatic radiation of a few THz, but with powers limited to tens of mW^29^, which limits their use in applications requiring high peak power.

The monochromaticity of the PDO may also be relevant to explaining observed narrowband radio-bursts from the solar corona^21^.

## Results

**Figure 2:**
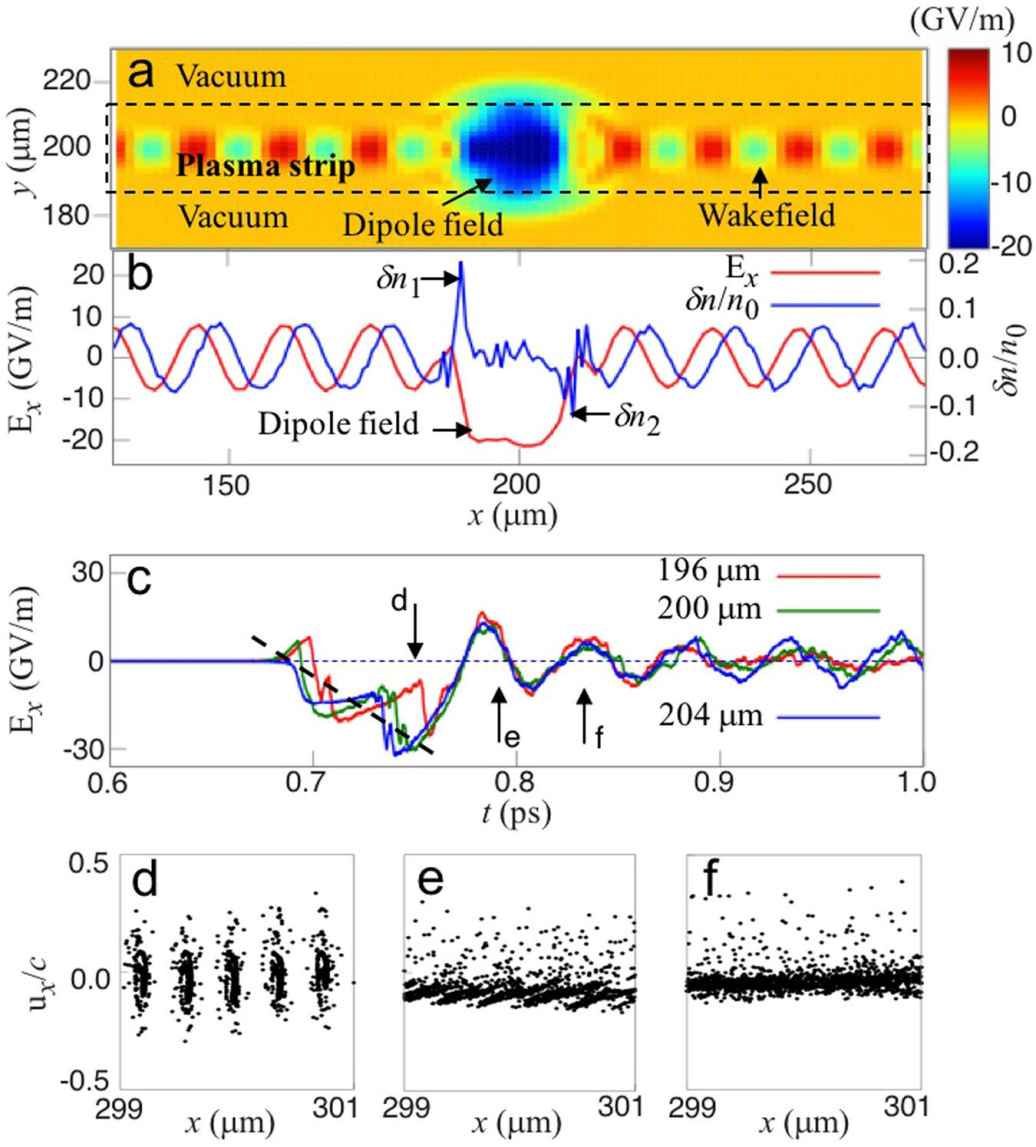
Displacement and release of trapped electrons from a 2D PIC simulation with normalized peak laser amplitudes *a*_0_ = 0.6 (where *a*_0_ = *eE*_0_/(*mcω*_0_), *ω*_0_ = 2*πc*/λ_0_ and the normalizing wavelength λ_0_ = 1 *μ*m), respective wavelengths λ_1_ = 0.8 *μ*m, λ_2_ = 0.76 *μ*m, duration *τ* = 30 fs for both laser pulses, and unperturbed plasma density *n*_0_ = 4.97 × 10^18^ cm^−3^. (**a**). The dipole field in 2D, (**b**). the perturbation of the electron density *δn*/*n*_0_ (blue), and the electric field (red) along the axis. (**c**) The electric field probed at three different positions inside the dipole in the axial direction (at y = 200 μm). (**d–f**). The phase portraits of the electrons close to the collision centre of the pulses, at *t* = 0.75, 0.8, and 0.83 ps, respectively.

The “displacement-release” process arising from colliding laser pulses is numerically demonstrated using two-dimensional (2D) particle-in-cell (PIC) simulations (Fig. 2). The laser pulses counter-propagate along the *x*-axis and collide head-on at the centre of a plasma slab, which is indicated by the rectangular region enclosed by dashed lines in Fig. 2a. As ion species, we considered He^2+^. During the dipole build-up and the subsequent oscillation, the motion of ions is negligible. The electron displacement yields a pair of oppositely-charged layers (Fig. 2b, blue), which produces a growing dipole field, indicated by the large blue spot near the centre of Fig. 2a and the red one in Fig. 2b. The displacement of the layers is comparable with the pulse length. As the micro-bunches of the trapped electrons (Fig. 2d) move with constant *v*_*ϕ*_ during the pulse collision, the dipole electric field *E*_di_(*t*) increases linearly over time (Fig. 2c, dashed line). The rapid oscillation at the bounce frequency *ω*_*b*_ = 2*ωa*_0_ during the linear growth confirms that the field growth is produced by trapped electrons, which produces an electrostatic dipole. Following the pulse collision, the dipole block undergoes relaxation-oscillation  at the plasma frequency (*t* > 0.75 in Fig. 2c). During this relaxation-oscillation period, the microbunches of electrons inside the dipole block stochastically merge^30^ to form a single super-bunch (Fig. 2e,f. More detail can be found in the Supplemental Section). The in-phase oscillations at different longitudinal positions inside the dipole block (Fig. 2c) confirm that the ensemble-averaged motion of electrons inside the dipole are locked in a single phase. This feature is distinct from regular wave-like motion of electrons, where their phases are locked in a periodic distribution, *i.e*. in a travelling wave. Note that the large charge separation is a result of coherent displacement of trapped electrons in multiple potential wells, which grow in number as the overlap between the laser pulses increases while the counter-propagating pulses progress through each other. As the dipole size is smaller than the radiation wavelength (2*πc*/*ω*_*p*_), the phase-locked current leads to coherent radiation, *i.e. I*_rad_ ∝ *N*^2^, where *N* is the number of electrons inside the dipole. In the following, we show that dipole oscillation can be indeed coupled to an electromagnetic radiation escaping into vacuum.

**Figure 3:**
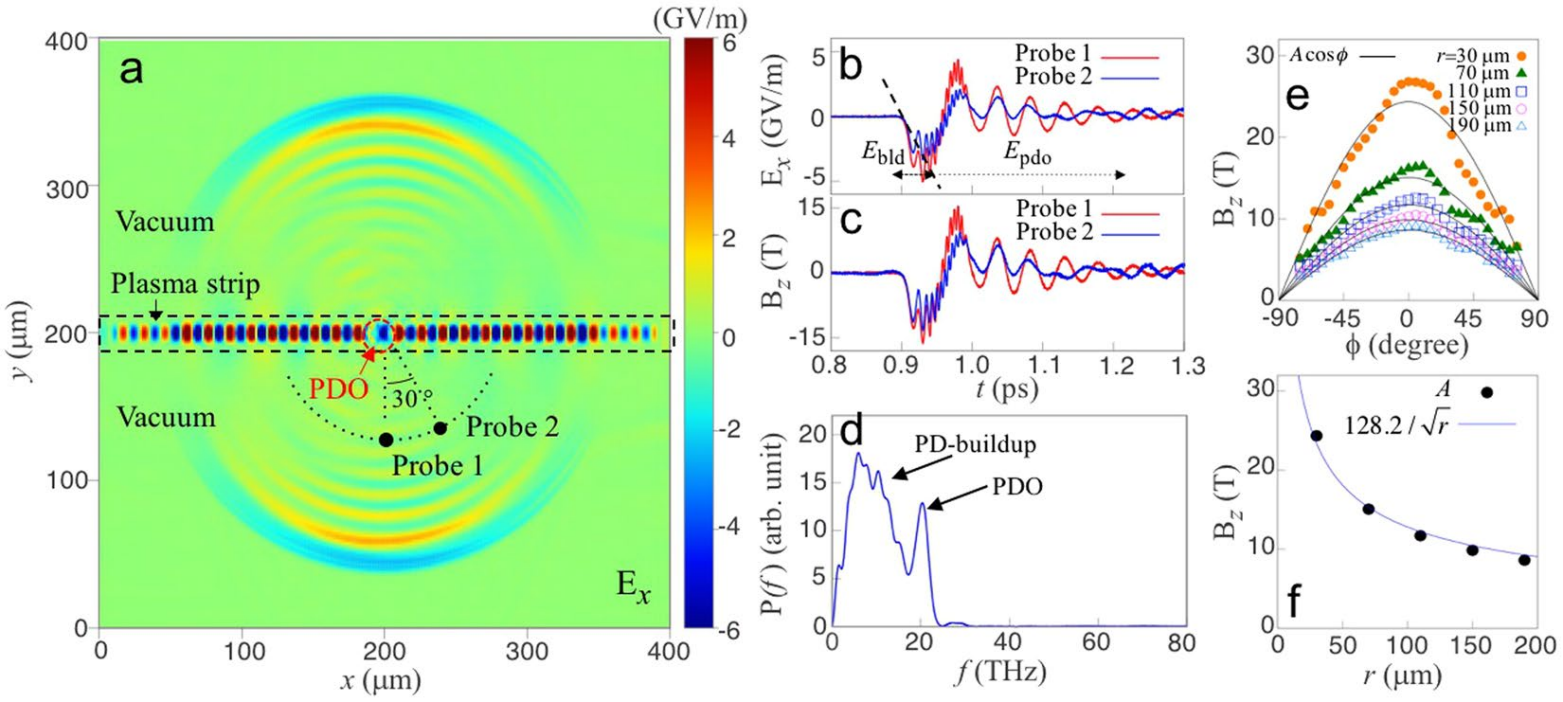
The radiation emitted, corresponding to the simulation of Fig. 2. (**a**) The *x*-direction electric field (*E*_*x*_) 0.54 ps after the pulse collision. The plasma is located in the region enclosed by the dashed lines. The plasma density is uniform along *x*, and trapezoidal in *y* with 10 *μ*m-ramps at both ends and a 20 *μ*m-plateau. Radiation fields are “measured” by virtual probes located at a distance of 70 *μ*m from the dipole centre, at 0° and 30°, respectively. (**b,c**). Evolution of the (*E*_*x*_) component of the electric field polarized in the *x*-*y*-plane, and *B*_*z*_, determined at the probe positions. (**d**) Power spectrum of *E*_*x*_ in (**b**). (**e**) The leading peak of *B*_*z*_ of the radiated pulse vs. emission angle. The solid lines are fitting curves to *A*cos*ϕ*. *ϕ* is the angle from the normal to the plasma strip and *A* is a fitting parameter, which corresponds to the peak amplitude of the radiated pulse. (**f**) Peak amplitude of *B*_*z*_ (i.e. *A*) vs. distance from the dipole centre.

The PDO presented in Fig. 2 shows that intense radiation is emitted with a spherical phase front (Fig. 3a). The peak amplitude of the radiation field reaches 5 GV/m, determined at 70 *μ*m from the centre of the dipole (Fig. 3b), and even larger at closer distances (*e.g*. >10 GV/m for *r* < 30 *μ*m, not shown). The in-phase oscillation of the fields determined at 0° and 30° (Probe 1 and 2 in Fig. 3a), respectively, indicates high transverse coherence of the emission. The initial increase in the radiation field (dashed line in Fig. 3b) originates from the increase in current during the PD build-up (Fig. 2c); during the PD build-up, as the current increases from zero to a certain value, *i.e. dI*/*dt* ≠ 0, the radiation is emitted. This is consistent with the time difference between the beginning of the field rising at the dipole centre (Fig. 2c) and at Probe 1 (Fig. 3b), which corresponds to the time taken for the radiation emitted from the dipole to reach Probe 1. Note that since the radiation field is much weaker than the driving laser field, the radiative energy loss during the dipole build-up does not greatly affect the build-up process, and is neglected in the simplified 1D model presented in the next section.

The power spectrum consists of two distinct parts: a very broad peak at low frequency and a quasi-narrowband peak at high frequency (Fig. 3d). The broad peak arises from radiation (*E*_bld_ in Fig. 3b) emitted by the current growth during the dipole build-up period, which has a typical radiation spectrum consistent with the transient current variation^23,24^. The quasi-narrowband peak is located at the plasma frequency (20 THz, in this case), implying that the PDO is radiating (*E*_pdo_ in Fig. 3b). The angular cosine-dependence (Fig. 3e) and radial decay $\propto 1/\sqrt{r}$ (Fig. 3f) are convincing evidence that the electromagnetic fields in the vacuum surrounding the plasma strip (Fig. 3a–c) arise from a 2D-dipole. The measured total energy of the emitted radiation, per unit lateral length (*i.e*., in *z*), is 0.4 J/mm, and the respective energy conversion efficiency is 2 × 10^−4^.

As the dipole oscillates in *x*-direction, the radiated electric field is linearly polarized in the *x*-*y*-plane, and accordingly the magnetic component is in the *z*-direction. Figure 3b and c demonstrate the temporal evolution of *E*_*x*_ (component of ${E}_{x}\hat{x}+{E}_{y}\hat{y}$) and *B*_*z*_.

**Figure 4:**
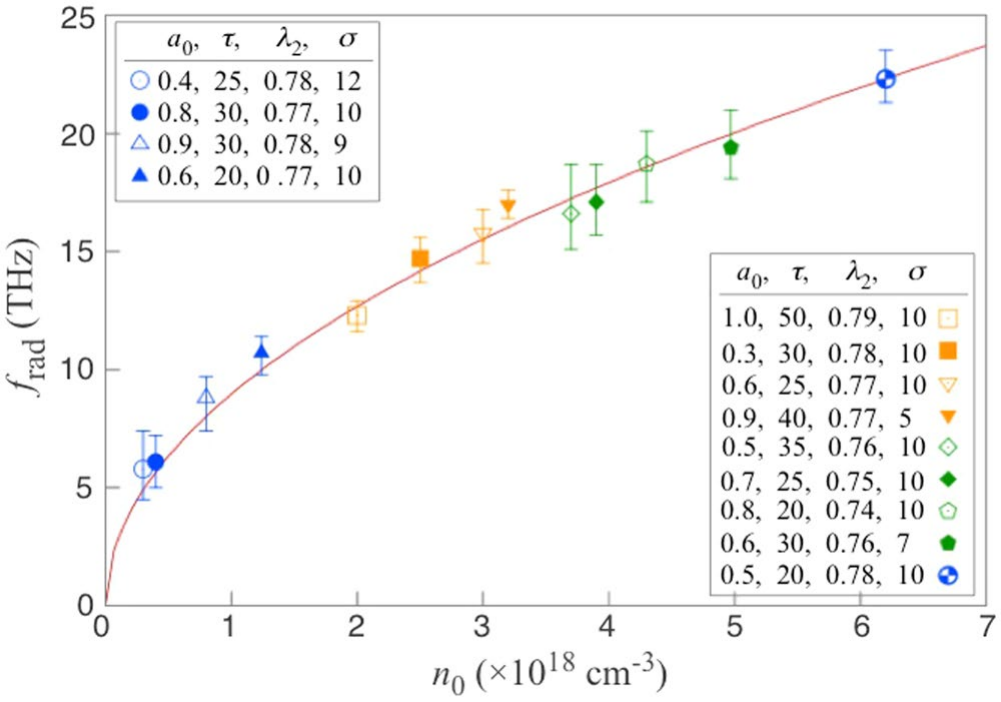
Radiation frequency, *f*_rad_ vs. plasma density, *n*_0_, of the plasma strip. The frequency of the radiation is determined at 100 *μ*m from the centre of the dipole. The error bars indicate the full width at half-maximum of the spectrum. The solid line is the plasma frequency as a function of the plasma density, *i.e*.${f}_{p}={(2\pi )}^{-1}\sqrt{{e}^{2}{n}_{0}/(m{\varepsilon }_{0})}$. In the figure legend, the pulse duration, *τ*, is given in femtoseconds. The wavelength of the second pulse, λ_2_, and its spot size, *σ*, are given in micrometers. The wavelength of the first laser pulse is fixed at 0.8 *μ*m.

The central frequency of the quasi-narrowband radiation coincides with the plasma frequency for a wide range of plasma densities (Fig. 4). It is clear from Fig. 4 that the parameters other than the plasma density, such as the driving pulse amplitude (*a*_0_), pulse duration (*τ*), spot size (*σ*) and the detuning (Δ*ω*, controlled via λ_2_) do not affect the radiation frequency. This provides further evidence that the radiation originates from the plasma oscillation. The spectral bandwidth (indicated by error bars) determined for the PDO-part of the emitted field (e.g. *E*_pdo_ in Fig. 3b) is comparable with the inverse of the pulse duration of the radiation. For the physical parameters used in the simulations (e.g., from Fig. 2d–f), the ion-electron collisional rate *ν*_*ei*_ is of order 10^10^ s^−1^, which is insignificant compared with the plasma frequency, and therefore can be neglected.

We note that when the laser pulses collide deeper in the bulk plasma instead of in a narrow strip, the maximum amplitude of the first oscillation of the radiation reduces as it is partially cut-off by the ambient plasma. We have observed in further 2D simulations that, when the plasma dipole is generated at the centre of a 200-*μ*m-wide plasma slab with constant density, the maximum amplitude of the radiation (30 *μ*m wavelength) escaping into vacuum is reduced by a factor of 2.5, compared with the case of the very narrow plasma strip. Dipole radiation from a narrow strip is similar to radiation by a localised surface plasmon. However, the radiation escaping through a wide bulk plasma requires a different explanation, which may be given by our concept of diffusing-growing field at the cut-off^31,32^.

In Fig. 3, the radiation from the bulk of the wakefield is negligible. This is consistent with the standard argument that an electrostatic plasma wave cannot be converted into an electromagnetic wave in plasma because their dispersion curves do not couple to each other and therefore precludes energy exchange (except at *k* = 0). Even when the boundaries of the plasma wave are interfaced with vacuum through a density taper, as in our case, the radiation from each electron is cancelled out by mutual destructive interference. Broadband emission is observed from the first bubble of the wakefield, which is negligible compared with PDO radiation. This electromagnetic wave is superficially similar to the bow-like wake wave^33,34^, or the conical radiation from filamented plasma^10,35^. High frequency harmonics from, for example, a relativistic electron spike^36^, have not been observed in our simulations. Radiation at the second harmonic (SH) (2*ω*_*p*_) is observed in some cases, though usually only when the driving pulse is strong, but its intensity is very weak compared with that of the fundamental radiation at *ω*_*p*_.

We note that the effects of ion motion on the generation of the dipole, and subsequent radiation, is negligible. Three cases with fixed ions, H*e*^2+^ and H^+^ yield virtually indistinguishable figures to Fig. 3.

## Analysis

**Figure 5:**
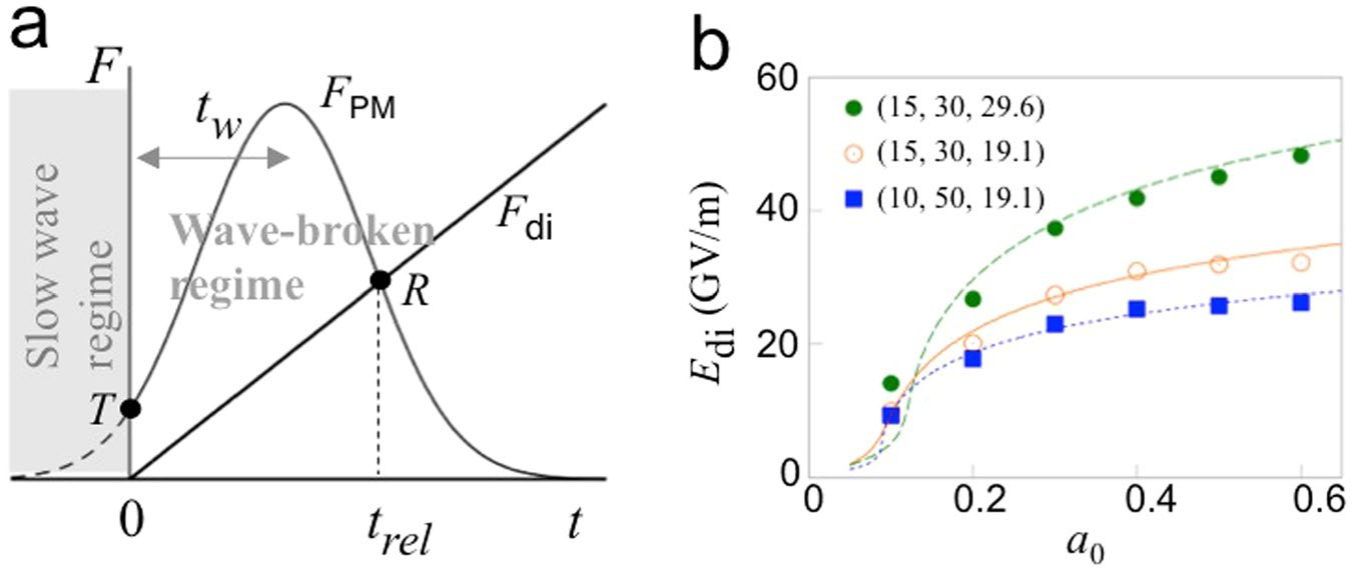
Force-balance model for calculating the maximum dipole field *E*_di_, and comparison with one-dimensional simulations. (**a**) Evolution of the ponderomotive (*F*_PM_) and electric restoring forces (*F*_di_). *T* and *R* are the wavebreaking threshold and release point of the dipole, respectively. (**b**) *E*_di_ vs. *a*_0_ from simulations (points) and Eq. (4) (lines). The numbers in parentheses are *f*_*p*_ (*ω*_*p*_/2*π*) (THz), the pulse duration *τ* (fs), and detuning Δ*f* (=Δ*ω*/2*π*) (THz).

The maximum displacement of electrons at the centre can be calculated using a force-balance model (Fig. 5a). When the normalized peak amplitude (*a*_0_) of the laser pulses is sufficiently high, the PM force increases more rapidly, at an early stage, than the restoring force of the dipole field, thereby enabling electron displacement. Subsequently, when the PM force drops below the restoring force, electron displacement halts and the electron dipole is released (Fig. 5a, point *R*) to oscillate. Given the time of the release, *t*_*rel*_ (Fig. 5a), the maximum dipole field is given by *E*_di_ = *St*_*rel*_, where *S* is the gradient of the dipole field growth. With the fraction of trapped electrons *α* (0 < *α* ≤ 1), from Gauss’s law,

1 $$S=\alpha \frac{e{n}_{0}}{{\varepsilon }_{0}}{v}_{\varphi }=\alpha \frac{e{n}_{0}}{{\varepsilon }_{0}}\frac{{\rm{\Delta }}\omega }{2k},$$

where −*e* is the charge of a single electron, ε_0_ is the vacuum permittivity, Δ*ω* = |*ω*_1_ − *ω*_2_|, and 2*k* = *k*_1_ + *k*_2_. This model predicts that, by increasing *a*_0_, Δ*ω*, the pulse duration *τ*, *t*_*rel*_ can be increased considerably, leading to very large *E*_di_. Note that the dipole field can be generated in a similar way by colliding *chirped* pulses, which is easy to realize in practice^37^. In this case, in general, the growth of the dipole field is not linear in time.

The growth of the dipole field due to the trapped electrons should be preceded by wavebreaking of the slow plasma wave, which is driven by the initial weak portion of the PM force (Fig. 5a). To determine *t*_*rel*_, we first calculate *t*_*w*_, which is defined by the time delay from the start of wavebreaking to that of the maximum PM force. Before wavebreaking, electrons are driven into harmonic motion by the PM force, *i.e*., $({\partial }_{t}^{2}+{\omega }_{p}^{2})\delta x=-{c}^{2}k{a}^{2}$, where *ω*_*p*_ is the plasma frequency, *a* the normalized amplitude of the laser pulses, and *δx* the electron displacement. For longitudinally Gaussian laser pulses, ${a}^{2}=\frac{1}{2}{a}_{0}^{2}\exp [-\mathrm{2(}t-{t}_{w}{)}^{2}/{\tau }^{2}]{e}^{i{\varphi }_{{\rm{PM}}}}+c\mathrm{.}c\mathrm{.}$, where *ϕ*_PM_ = 2*kx* − Δ*ωt*. Note that wavebreaking occurs at *t* = 0. With $\delta x=\frac{1}{2}\hat{x}{e}^{i{\varphi }_{{\rm{PM}}}}+{\rm{c}}\mathrm{.}{\rm{c}}\mathrm{.}$ (slow plasma wave), the equation of motion yields

2 $$\frac{{\partial }^{2}\hat{x}}{\partial {t}^{2}}+{\omega }_{p}^{2}\hat{x}=-c\omega {a}_{0}^{2}\exp \,[-\frac{\mathrm{2(}t-{t}_{w}{)}^{2}}{{\tau }^{2}}]\mathrm{.}$$

When $|\hat{x}(t=\mathrm{0)| > 1/(}{k}_{1}+{k}_{2})$, wavebreaking occurs due to the change in ordering of electrons^38^. In Eq. (2), we assume ${\partial }_{t}\gg {\rm{\Delta }}\omega$ for rapidly growing $\hat{x}$ (verified by PIC simulations), which is opposite to the regular assumption of the slowly-varying envelope, *e.g*., Ref.^39^. $\hat{x}\,(t=\mathrm{0)}$ calculated from Eq. (2) and the wavebreaking condition lead to

3 $$\frac{8}{{\omega }^{2}{\tau }^{2}{a}_{0}^{2}}\simeq \frac{{e}^{-2{\xi }^{2}}}{{\xi }^{2}+{\omega }^{2}{\tau }^{2}\mathrm{/16}}\mathrm{\ ,}\,\xi =\frac{{t}_{w}}{\tau }\mathrm{.}$$

**Figure 6:**
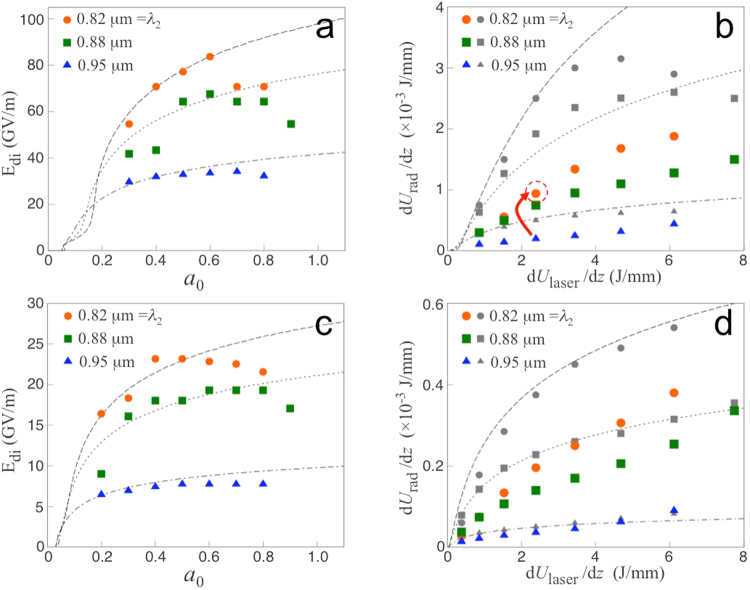
The maximum dipole field *E*_di_ at the dipole centre, the initial energy of the dipole per unit lateral length *dU*_di_/*dz* and total energy of the radiation per unit lateral length *dU*_rad_/*dz*, as functions of the peak amplitude of the laser pulse *a*_0_ or total energy of the driving laser pulse per unit lateral length *dU*_laser_/*dz*, obtained from two-dimensional simulations. The wavelength of the first laser pulse is λ_1_ = 1 *μ*m. Three different cases of detuning with λ_2_ = 0.82 *μ*m (circles), 0.88 *μ*m (squares) and 0.95 *μ*m (triangles) have been simulated. (**a**,**c**) *E*_di_ vs. *a*_0_ for *ω*_*p*_/2*π* = 20 and 10 THz, respectively. Theoretical curves from Eq. (4) fit the simulation data best with *α* = 0.7, 0.8 and 1.0 from the top to the bottom. (**b**,**d**) *dU*_di_/*dz* (lines and grey markers) and *dU*_rad_/*dz* (coloured markers) vs. *dU*_laser_/*dz* for *ω*_*p*_/2*π* = 20 and 10 THz, respectively. Theoretical curves from Eq. (5) fits the simulation data best with *R* = 0.6 (**b**) and 0.8 (**d**).

The release time *t*_*rel*_ can be obtained by balancing the ensemble-averaged PM force with the restoring force (−*eE*_di_). From *E*_di_ = *St*_*rel*_,

4 $$\alpha \frac{{\omega }_{p}^{2}}{2{\omega }^{2}}{\rm{\Delta }}\omega {t}_{rel}=\eta \,\frac{{a}_{0}^{2}}{\sqrt{1+{a}_{0}^{2}\mathrm{/2}}}\exp [-\frac{\mathrm{2(}{t}_{rel}-{t}_{w}{)}^{2}}{{\tau }^{2}}],$$

where *η* is from the ensemble average of the PM force exerted on electrons scattered inside each PM potential segment. The ensemble average is equivalent to a time-average during one bounce cycle (*i.e*., *x* = *A*sin *ω*_*b*_*t* + *x*_*c*_, where *A* and *x*_*c*_ are the amplitude and centre of the bouncing motion, respectively), leading to *η* = *J*_0_(2*kA*) sin 2*kx*_*c*_, where *J*_0_ is the zero’th order Bessel function. From *A* ≤ λ_*b*_/2 = *π*/2*k* and sin 2*kx*_*c*_ ≤ 1, *η* ≤ 0.3. *E*_di_ from numerical solutions of Eq. (4) along with *η* = 0.3 (and with *α* = 1) agrees well with 1D PIC simulation data for a diverse range of parameters (Fig. 5b and the Supplemental Section). For electrons with non-central initial position *x*_0_ (zero is the collision centre), the right-hand-side of Eq. (4) should be multiplied by $\exp [-2{x}_{0}^{2}/(c\tau {)}^{2}]$. In the calculation of *E*_di_, we neglect the coupling of the radiation field, since the driving laser field is dominant over the radiation field during the build-up of the dipole. Figure 6a and c show a comparison between *E*_di_ obtained from Eq. (4) and that from two-dimensional simulations. With the fraction of trapped electrons *α* adjusted to 0.7 ~ 1.0, the theoretical curves are in reasonable agreement with the simulation data. Note that *α* tends to decrease as the detuning increases, since electrons are more strongly thermalised by the high phase velocity for a large detuning and are de-trapped more readily from the ponderomotive potential.

To estimate the radiation energy, we assume that a major part of the initial dipole field energy *U*_di_ is converted to radiation energy *U*_rad_. In 2D, the initial field energy per unit length in *z* (*dU*_di_/*dz*) contained in the dipole region over [−*τ*,*τ*] × [−*σ*,*σ*] (*τ* and *σ* are the pulse duration and spot radius of the laser pulses, respectively) is

5 $$\frac{d{U}_{{\rm{d}}{\rm{i}}}}{dz}=R\frac{{\varepsilon }_{0}}{2}{\int }_{-\tau }^{\tau }cdt{\int }_{-\sigma }^{\sigma }dy{E}_{{\rm{d}}{\rm{i}}}^{2}(y).$$

In Eq. (5), we assume that the transverse motion of electrons is negligible and *E*_di_(*y*) is determined by the one-dimensional equation (4), where *a*_0_ is the field strength at each transverse position *y*, i.e. *a*_0_(*y*) ∝ exp[−*y*^2^/*σ*^2^] for a transversely Gaussian laser pulses. As the theoretical value of *E*_di_ tends to overestimate the dipole field outside the central region, we have employed a phenomenological reduction factor *R* in Eq. (5). With *R* ≥ 0.6, Eq. (5) agrees with results of 2D PIC simulations for *f*_*p*_ = 20 and 10 THz (grey markers in Fig. 6b and d). Since the electrons located far from the dipole centre are not quite in phase with the central electrons, not all the initial dipole energy *U*_di_ contained over [−*τ*,*τ*] × [−*σ*,*σ*] is necessarily converted to the radiation energy *U*_rad_. Instead Eq. (5) rather gives the upper limit to the radiation energy. Figure 6 (b) and (d) demonstrate that *U*_rad_ is measured to be about 50 percent or more of *U*_di_. However, when *U*_di_ is calculated over a smaller range, such as over the full-width at the half-maximum, it becomes comparable to or even smaller than *U*_rad_.

The total radiation energy in 3D can be estimated by multiplying Eq. (5) by the lateral dimension (*z*-length) of the dipole. Assuming a highly elliptical laser beam^40,41^, e.g., 10 *μ*m × 1 mm laser spot, the total energy of the dipole radiation is expected to reach a few millijoules (Fig. 6b). The theoretical maximum efficiency of the energy conversion, $\epsilon ={U}_{{\rm{r}}{\rm{a}}{\rm{d}}}/{U}_{{\rm{l}}{\rm{a}}{\rm{s}}{\rm{e}}{\rm{r}}}\simeq 0.1\frac{{\omega }_{p}}{\omega }{\beta }_{\varphi }{\omega }_{p}\tau$, where *β*_*ϕ*_ = Δ*ω*/(2*ck*) (see the Supplemental Section for the derivation). This scaling indicates that a higher efficiency can be obtained by increasing the detuning and the pulse duration. Figure 6b shows that the increased detuning leads to a rise in the efficiency by an order of magnitude, up to 0.4 × 10^−3^ (arrow), which is very high compared with other mJ laser-based THz systems. Even higher efficiencies beyond 10^−2^ are expected with further optimization (see the Supplemental Section). Note that the only available method, other than laser-based ones, for obtaining mJ, narrowband THz pulses at tens of terahertz is the FEL^42^, which has an extensive user community investigating, amongst other topics, hydrogen bonding or inter-molecular vibrational modes^43,44^.

## Discussion and Conclusion

In contrast to broadband, weakly-tuneable, microjoule THz bursts generated from laser-solid interactions^23,24^, concentrating millijoule energies into a narrowband spectrum with high tunability has significant advantages in applications that require strong terahertz fields^25–28,43,44^. The PDO may be relevant to emission from very different plasma systems, such as ejection of energetic electron beams (*e.g*., by magnetic reconnection^45^), which is relevant to astrophysical plasma (e.g., in the solar corona^16^), pairs of colliding electron beams could possibly generate dipole moments by trapping electrons in plasma waves, which grow from streaming instabilities. Similar dipole radiation may have been produced (but overlooked) in laser-solid interactions^14,23^, where the reflected pulse overlaps the incident pulse obliquely in the underdense plasma plume ejected from the solid surface. In this system, the Doppler-shift caused by the moving reflection surface of the plasma or a chirp would provide the reflected and incident pulses with the necessary frequency-detuning for a dipole to occur when the laser pulse has passed through itself on reflection. The mechanism of radiation here is distinct from ref.^31^, where an external magnetic field is used to deflect electrons. Coalescence of two plasmons^4,17^, which generate second harmonic radiation, is also different from PDO, where the fundamental harmonic radiation is dominant. An oscillating plasma surface^46–48^ is also a localized plasma oscillation, but exists only on a relatively steep plasma-vacuum interface. Moreover, it requires an incident wave for high-harmonic generation, which is distinct from our self-emitting, embedded PDO. Particle trapping by counter-propagating pulses has been studied previously^39,49–53^, but a localized PDO and ensuing radiation were not perceived.

In conclusion, we have investigated a new mechanism of radiation emission from plasma oscillations, which might be relevant to diverse radiation phenomena that are unexplained or may have been overlooked. Practically, the millijoule-THz from PDO would stimulate novel terahertz-based non-linear sciences.

## Methods

### Simulation code

For the particle-in-cell simulations, we used the ‘cplPIC’ code, which employs the standard Yee-mesh-based field solver^54^, Villasenor-Buneman charge-conserving scheme for current calculation^55^ and Boris mover for particle motion^56^. The ‘cplPIC’ code has been verified via diverse simulation studies in laser wakefield electron acceleration^57^, target-normal-sheath ion acceleration^58^, shock-ion-acceleration^59^, terahertz emission from a magnetized plasma^31^, terahertz emission from density gradient^7^ and Raman amplification^1^ (the envelope module was used for this study).

### Simulation parameters

In the one-dimensional simulations, the mesh size and time step are Δ*x* = 4 × 10^−8^ m and Δ*t* = 1.3 × 10^−16^ s, respectively. The number of simulation particles per cell is 5. In the two-dimensional simulations, the mesh size in each direction is Δ*x* = 5 × 10^−8^ m and Δ*y* = 2 × 10^−7^ m, respectively, and the time step is Δ*t* = 1.5 × 10^−16^. The numbers of simulation particles per cell for electrons and He^2+^ ions are 3~5 and a half of that, respectively, depending on the plasma density. We checked that EPOCH 2D code with the same parameters yields a similar radiation pattern and strength (not shown).

### Data availability

Data associated with research published in this paper is available at 10.15129/ca8c9bbd-24e4-41a8-b022-434d8b6e587f.

## Electronic supplementary material

supplemental material

Simulation of 1D dipole generation
