# Supplementary material for: High-Energy, Short-Duration Bursts of Coherent Terahertz Radiation from an Embedded Plasma Dipole
Source: Sci Rep. 2018 Jan 9;8:145. doi: 10.1038/s41598-017-18399-3 (PMC5760715; doi:10.1038/s41598-017-18399-3)
Supplement: Supplementary file 1 — supplemental material [file 41598_2017_18399_MOESM1_ESM.pdf]

# Supplemental material: High-Energy, Short-duration Bursts of Coherent Terahertz Radiation from an Embedded Plasma Dipole

Kyu Been Kwon,[1] Teyoun Kang,[1] Hyung Seon Song,[1] Young-Kuk Kim,[1] Bernhard Ersfeld,[2] Dino A. Jaroszynski,[2,*] and Min Sup Hur[1,†]

*[1]School of Natural Science, UNIST, 50 UNIST-gil, Ulju-gun, Ulsan, 44919, Korea*

*[2]Department of Physics, Scottish Universities Physics Alliance and University of Strathclyde, Glasgow G4 0NG, UK*

[*]Electronic address: `D.A.Jaroszynski@strath.ac.uk`

[†]Electronic address: `mshur@unist.ac.kr`

In this supplemental material, we present **I.** derivation of $t_w$ in Eq. (3), **II.** comparison of Eqs. (1) and (3) with one-dimensional PIC simulations and additional simulation data for Eq. (4), **III.** effects of stochastic heating on PDO and **IV.** optimal efficiency of the energy conversion.

## I. DERIVATION OF $t_w$ IN EQ. (3)

The ponderomotive (PM) force is given by

$$f_{pm} = -mc\omega\frac{a^2}{\bar{\gamma}}\left(\frac{1}{2}e^{2ikx-i\Delta\omega t} + c.c.\right) \;, \quad a = a_0 \exp\left[-\frac{(t-t_w)^2}{\tau^2}\right], \tag{s-1}$$

where the time-averaged Lorentz factor $\bar{\gamma} = \sqrt{1+\bar{a^2}}$ and $\omega$ is the angular frequency of the laser pulse ($\omega \simeq \omega_1 \simeq \omega_2$). We neglect the relativistic effect, that is, $\bar{\gamma} \simeq 1$. This is possible, since the phase velocity of the plasma wave driven by the laser beating is very low ($v_\phi \ll c$), and accordingly, the wavebreaking threshold is very low. Hence, near or below the wavebreaking, $a \ll 1$, leading to $\bar{\gamma} \simeq 1$. In Eq. (s-1), $t_w$ is the time from the wavebreaking to the peak of the PM force (see Fig. 4a in the main article). Note that Eq. (s-1) is arranged so that the PM force reaches the wavebreaking threshold at $t = 0$. The wavebreaking is not exactly the same phenomenon as the electron trapping. However, we assume that the electron trapping follows the wavebreaking with almost no delay, since the plasma wave grows very rapidly.

In the Lagrangian description, the displacement ($\delta x$) of an electron fluid element is governed by

$$\frac{\partial^2 \delta x}{\partial t^2} + \omega_p^2 \delta x = -c\omega a^2\left(\frac{1}{2}e^{2ikx_0-i\Delta\omega t} + c.c.\right). \tag{s-2}$$

Note that $x_0$ (initial position of the electron) is used instead of $x$ (current position of the electron) in the argument of the oscillating function in the right-hand-side. This is reasonable, since we consider a small amplitude oscillation, i.e. $k\delta x \ll 1$, where $x = x_0 + \delta x$. We write the electron displacement $\delta x$ by

$$\delta x = \frac{1}{2}\hat{x}(t)e^{2ikx_0-i\Delta\omega t} + c.c., \tag{s-3}$$

where $\hat{x}$ is the time-varying amplitude. Usually, $\hat{x}$ is slowly-varying compared to $\Delta\omega^{-1}$, but in our case, the growth of $\hat{x}$ is much faster than the beat oscillation, i.e. $\Delta\omega \ll \partial_t$, at

least below the wavebreaking threshold. This point has been verified throughout numerous simulations (not shown). Hence, Eq. (s-2) leads to an amplitude equation as follows.

$$\frac{\partial^2 \hat{x}}{\partial t^2} + \omega_p^2 \hat{x} = -c\omega a^2. \tag{s-4}$$

Note that $e^{i\phi_{\rm PM}}$ has been cancelled out from both sides of Eq. (s-4). Green function of the harmonic operator in the left-hand-side is given by $G(t,t') = \frac{1}{\omega_p}\sin\omega_p|t-t'|$. Then we calculate the evolution of $\hat{x}$ until the wavebreaking ($t=0$) as follows.

$$\begin{aligned}\hat{x}(t=0) &= -\frac{c\omega}{\omega_p}\int_{-\infty}^{0} a^2 \sin\omega_p t\; dt = -\frac{c\omega}{\omega_p}a_0^2\int_0^{\infty}\exp\left[-\frac{2(t+t_w)^2}{\tau^2}\right]\sin\omega_p t\; dt \\ &= -\frac{c\omega}{\omega_p}a_0^2\exp\left[-\frac{2t_w^2}{\tau^2}\right]\int_0^{\infty} e^{-\beta t^2-\gamma t}\sin bt\; dt,\end{aligned} \tag{s-5}$$

where

$$\beta \equiv \frac{2}{\tau^2}\ ,\quad \gamma \equiv \frac{4t_w}{\tau^2}\ ,\quad b \equiv \omega_p.$$

Using 3.897 of Gradshteyn and Ryzhik "*Table of Integrals, Series, and Products*" the 7th edition, with $\Re\beta > 0$, the integral in (s-5) becomes

$$\begin{aligned}&\int_0^{\infty} e^{-\beta t^2-\gamma t}\sin bt\; dt \\ &= -\frac{i}{4}\sqrt{\frac{\pi}{\beta}}\left\{\exp\frac{(\gamma-ib)^2}{4\beta}\left[1-\Phi\left(\frac{\gamma-ib}{2\sqrt{\beta}}\right)\right]-\exp\frac{(\gamma+ib)^2}{4\beta}\left[1-\Phi\left(\frac{\gamma+ib}{2\sqrt{\beta}}\right)\right]\right\},\end{aligned} \tag{s-6}$$

where $\Phi$ is the error function. The arguments of the exponential function and the error function in Eq. (s-6) are rewritten by

$$\begin{aligned}\frac{\gamma \pm ib}{2\sqrt{\beta}} &= \frac{\sqrt{2}t_w}{\tau} \pm i\frac{\omega_p\tau}{2\sqrt{2}} = T_w \pm iW_p\ ,\quad T_w \equiv \frac{\sqrt{2}t_w}{\tau}\ ,\quad W_p \equiv \frac{\omega_p \tau}{2\sqrt{2}} \\ \frac{(\gamma\pm ib)^2}{4\beta} &= (T_w^2 - W_p^2) \pm 2iT_wW_p = (T_w^2-W_p^2) \pm i\omega_p t_w.\end{aligned} \tag{s-7}$$

The magnitude of this argument is larger than unity, i.e. $|T_w \pm W_p| > 1$. In this range, the asymptotic form can be used for the error function: $\Phi(z) \simeq 1 - \frac{e^{-z^2}}{\sqrt{\pi}z}\left(1-\frac{A}{z^2}\right)$, leading to

$$\Phi(\pm) \simeq 1 - \frac{e^{-T_w^2+W_p^2}e^{\mp i\omega_p t_w}}{\sqrt{\pi}(T_w^2+W_p^2)}(T_w \mp iW_p)\left(1-\frac{A(T_w\mp iW_p)^2}{(T_w^2+W_p^2)^2}\right), \tag{s-8}$$

where $A = 1/2$. Using this expansion, the integral (s-6) becomes

$$\int \sim \;= \frac{\tau}{2\sqrt{2}}\frac{W_p}{T_w^2+W_p^2}\left[1 - A\frac{3T_w^2-W_p^2}{(T_w^2+W_p^2)^2}\right]. \tag{s-9}$$

Then finally, with $\xi \equiv t_w/\tau$ in Eq. (s-5),

$$\hat{x} = -\frac{c\omega}{\omega_p} a_0^2 \frac{\omega_p \tau^2}{16} \frac{e^{-2\xi^2}}{\xi^2 + \omega_p^2\tau^2/16} \left(1 - \frac{A}{2} \frac{3\xi^2 - \omega_p^2\tau^2/16}{(\xi^2 + \omega_p^2\tau^2/16)^2}\right). \tag{s-10}$$

From [J.M. Dawson, Phys. Rev. **113**, 383 (1959)], the wavebreaking condition is given by

$$|\hat{x}_1| \geq \frac{1}{k_b} = \frac{1}{2k}, \tag{s-11}$$

where the wavenumber of the beat $k_b = k_1 + k_2 = 2k$. Then equations (s-10) and (s-11) yield

$$\frac{8}{\omega^2\tau^2 a_0^2} = \frac{e^{-2\xi^2}}{\xi^2 + \omega_p^2\tau^2/16} \left(1 - \frac{A}{2} \frac{3\xi^2 - \omega_p^2\tau^2/16}{(\xi^2 + \omega_p^2\tau^2/16)^2}\right), \quad \xi = \frac{t_w}{\tau}. \tag{s-12}$$

In the main text, we calculated just up to the leading order term by setting $A = 0$.

## II. COMPARISON OF EQS. (1), (3) AND (4) WITH ONE-DIMENSIONAL PIC SIMULATIONS

Here we present one-dimensional PIC simulation data, which support Eqs. (1), (3) and (4).

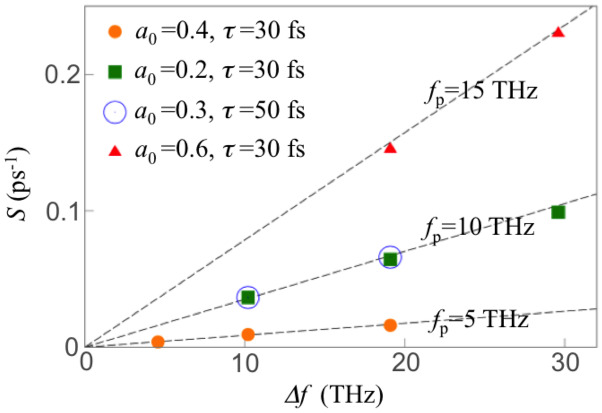


FIG. 1s: The slope $S$ of the linear field growth of the dipole field vs. the frequency difference of the laser pulses, for various plasma densities (given by $n_0 = 4\pi^2 f_p^2 m\epsilon_0/e^2$), the normalized pulse amplitude $a_0 = eE_0/mc\omega$, and the pulse duration $\tau$. The slope of the straight lines are from Eq. (1) of the main article.

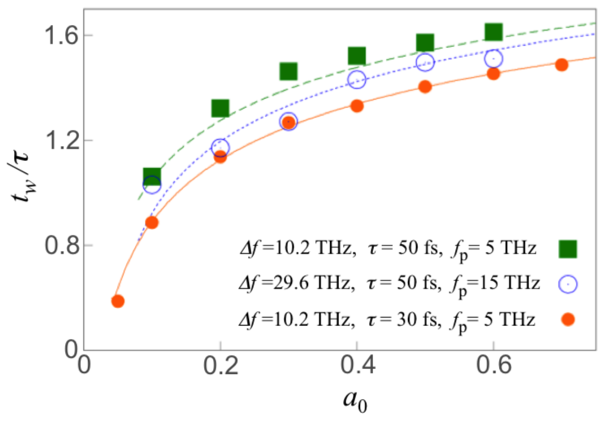


FIG. 2s: $t_w$ vs. $a_0$ for various frequency difference $\Delta f$ (THz) of the laser pulses, the pulse duration $\tau$ (fs), and the plasma density $n_0$. In the legend, $n_0$ is represented by the plasma frequency $f_p = (2\pi)^{-1}\sqrt{e^2 n_0/m\epsilon_0} \times 10^{-12}$ (THz). The theoretical curves are from Eq. (3) of the main article.

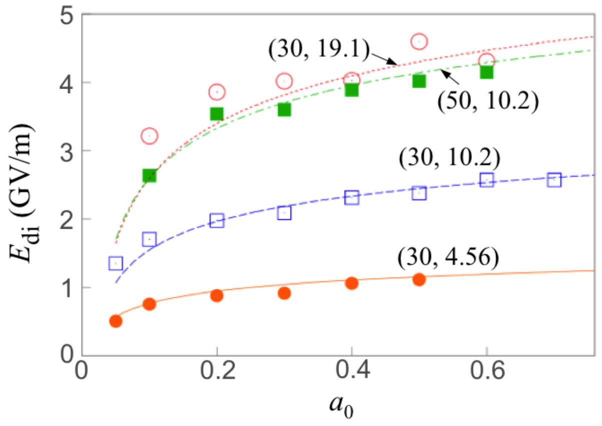


FIG. 3s: $E_{\rm di}$ vs. $a_0$ for $f_p = 5$ THz, and various $\Delta f$ and $\tau$. The numbers inside the parentheses represent $\tau$ (fs) and $\Delta f$ (THz). The theoretical curves are from Eq. (4) of the main article, with $\alpha = 1$ and $\eta = 0.3$.

## III. EFFECTS OF STOCHASTIC PHASE MIXING

It is known that the electron plasma wave driven by counter-propagating laser pulses is prone to particle trapping and subsequent stochastic phase mixing and heating. This is mainly because the phase velocity of the plasma wave is very low ($v_\phi \sim \Delta\omega/2k \ll c$), and

thus, the wavebreaking limit is too low to keep the bounded motion of electrons. Since the stochastic heating disrupts the coherent structure of the plasma wave of wavenumber $2k$ ($= k_1 + k_2$), it is a negative factor in constructing electron density gratings, Raman backward pulse amplification, and other applications using the slow wave generated by counter-propagating pulses. In contrast, the phase-mixing does not affect the coherency of the PDO. Indeed, the phase-mixing is a favorable part in generating a coherent PDO; ideally, the dipole-block should be quasi-neutral except at both edges. This can be accomplished as the short-wavelength micro-bunches inside the dipole-block are merged by the stochastic phase-mixing. The merged bunches generate a single, a-few-mircon-long super-bunch (named a dipole-block or an electron-block), which oscillates due to the regular plasma oscillation mechansim.

The procedure described above can be seen from 1D PIC simulations. Figure 4s represents the electron density after the pulse collision is finished. Due to the stochastic phase mixing and randomized motion of the electrons, the density profile looks very noisy. However, by filtering out the high-frequency noise, a very clear charge-separated density profile (blue line) comes out. The length of the charge-separated region is comparable to the pulse width, which includes several $2k$-wavelengths.

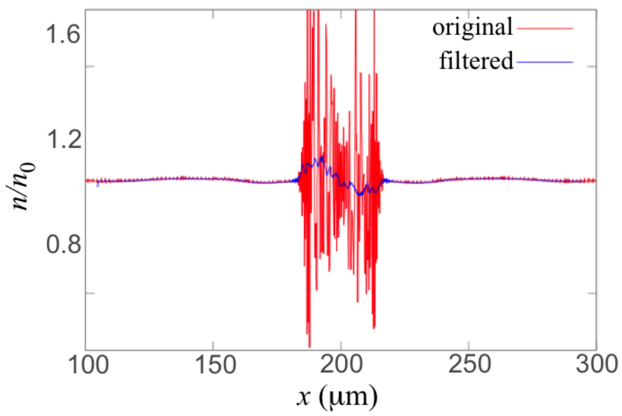


FIG. 4s: The original and high-frequency-filtered profiles of the electron density after the pulse collision. The parameters of the 1D PIC simulation are $a_0 = 0.1$, $\tau = 30$ fs, $\lambda_1 = 1$ $\mu$m, $\lambda_2 = 0.967$ $\mu$m, and $n_0 = 3.125 \times 10^{17}$ cm$^{-3}$. The length of the simulation domain is 400 $\mu$m. The density was captured at $t = 870$ fs.

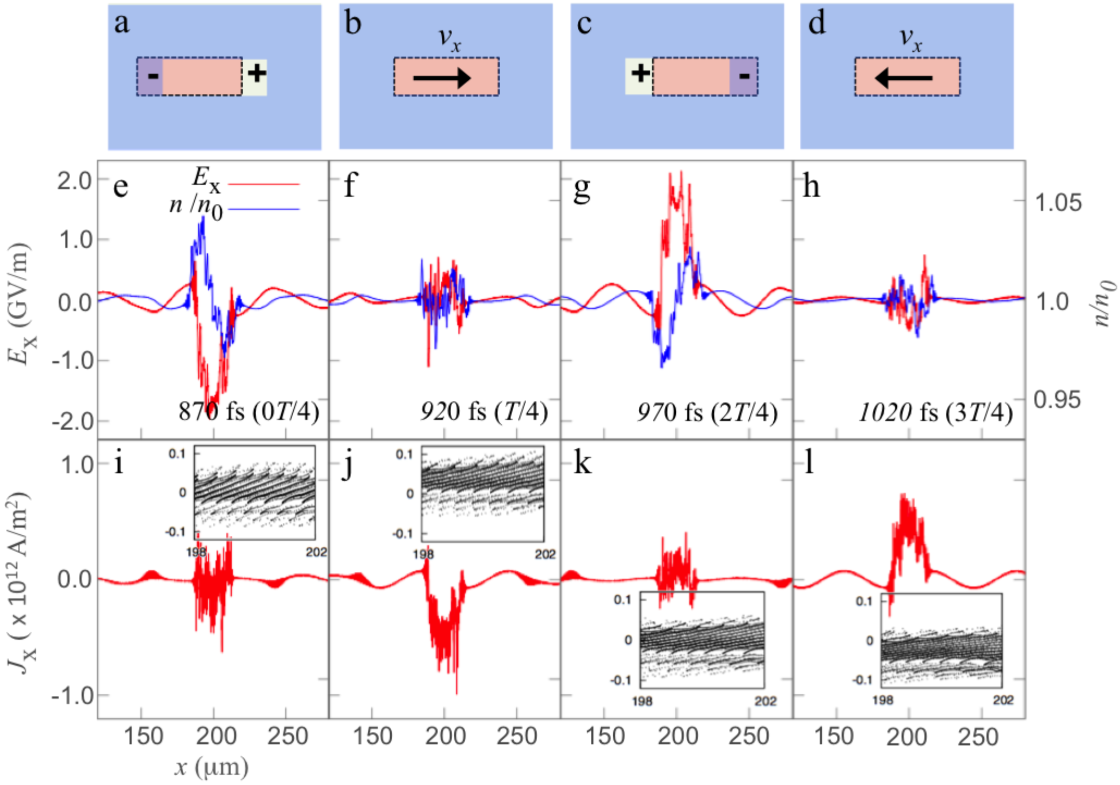


FIG. 5s: (a-d) Schematic figure of the dipole-block oscillation. The boxes enclosed by dashed lines represent the dipole electron block. (e-h) Corresponding longitudinal electric field ($E_x$), electron density ($n/n_0$), and (i-l) longitudinal electric current density ($J_x$), which was captured every $T/4$, where $T$ is the period of the plasma oscillation. The insets in (i-l) represent the electron phase space, $u_x/c$ vs. $x$. The simulation parameters are the same as those in Fig. 4s.

Figure 5s demonstrates that the electron-block oscillates in a single-object-like manner. The separated charge and the corresponding longitudinal electric field alternate every 200 fs (5 THz), which is the period of the plasma oscillation for the given plasma density. The current density $J_x$ also oscillates out-of-phase by 90 degree, due to the relation $J_x = \epsilon_0 \partial_t E_x$. The insets in Fig. 5s (i-l) are the $u_x/c - x$ space of the electrons over a region around the dipole-centre. When the current density is at its negative (positive) maximum [(j) and (l)], the centre of the particle velocity distribution is apparently positive (negative) [insets of (j) and (l)]. When the current density is close to zero [(i) and (k)], the velocity distribution is centered roughly at zero [insets of (i) and (k)]. These phase figures show that the thermalized electrons move back and forth as a single super-bunch.

We have also traced the temporal evolution of $J_x$ at different positions apart from the centre of the oscillating block by 4 $\mu$m (this is rough span of the oscillating block) (Fig. 6s). Figure 6s clearly demonstrates that, despite the random motion of the heated electrons, the averaged velocity (that is, the current density $J(x) = -e\int vf(x,v)dv = -en\bar{v}$, where $f(v)$ is the distribution function) is locked in-phase over the region of the laser-pulse overlap. Furthermore, the negligibly small high-frequency noise generated by the random motion in Fig. 6s (b) indicates that the random part of the electron motion does not contribute to the radiation emission.

From Figs. 5s and 6s, it is evident that $J(x)$ is in-phase over the dipole-block. Because the dipole size is smaller than (or, at most, comparable to) the radiation wavelength, the radiation emitted from $J(x)$ over the dipole superposes constructively. As $J \propto n$, where $n$ is the plasma density, the radiation intensity $I_{\rm rad} \propto N^2$, where $N = \int_{\rm dipole} nd^3x$, i.e. the number of electrons included in the dipole-block. This is a typical characteristic of a coherent radiation source.

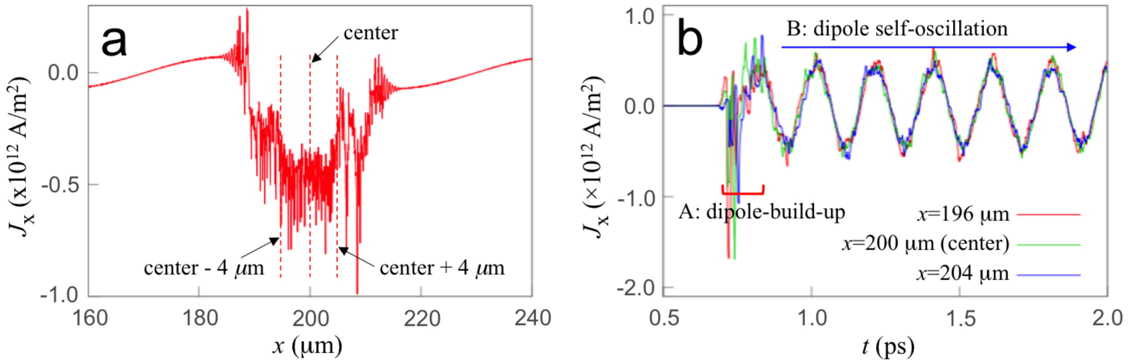


FIG. 6s: Evolution of the electric current density at the centre of the pulse collision (i.e. centre of the dipole), and $\pm 4$ $\mu$m from the centre. (a) The current density at $t = 960$ fs. The red dashed-lines represent the positions, where $J_x$ is measured as a function of time. (b) Oscillation of $J_x$ in the time-domain, showing excellent coherence of $J_x$ over different positions inside the dipole-block. 'A' represents the period of the dipole-build-up during the pulse collision, and 'B' represents the sustained self-oscillation of the dipole due to the plasma oscillaiton mechanism.

We can exclude the possibility of breaking the coherence by thermal Doppler broadening. From Figs. 7s (b) and (d), it is found that the $y$-directional velocity is very low. This indicates that in the collisionless regime as in our case, the relatively high longitudinal ($x$)

kinetic energy does not transfer significantly to the transverse ($y$) kinetic energy during the plasma oscillation. As the thermal spread in $y$-direction is small, the thermal spectral broadening of the radiation, which is emitted mostly in $y$-direction, should be negligible. The estimated broadening is about 0.5 percent, which is very small compared with the inherent spectral width of the PDO-radiation.

Because the PDO is not damaged by the phase-mixing, the driving pulse intensity needs not be kept low to preserve the slow wave structure. This is another advantage of the PDO over other oscillation mechanisms.

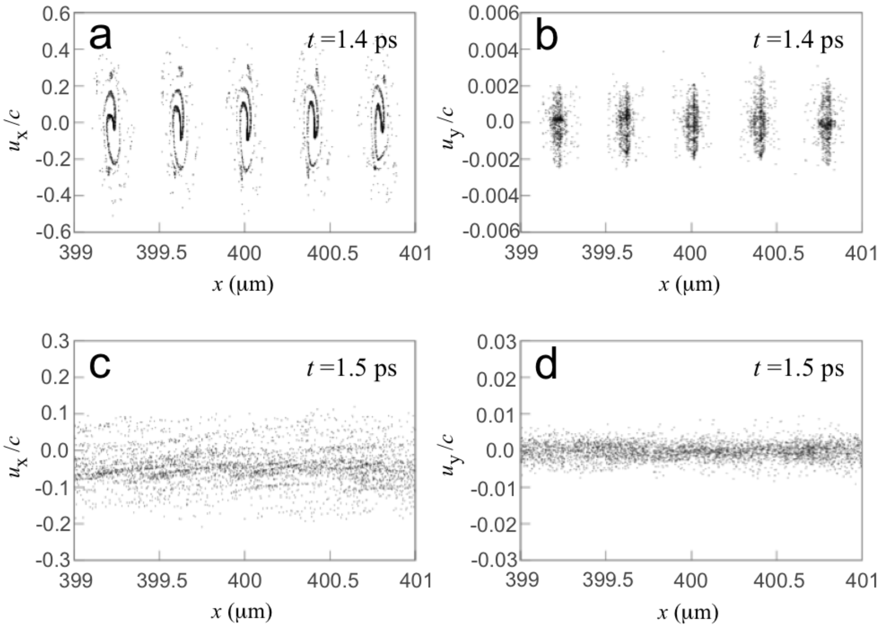


FIG. 7s: The phase portraits of the electrons around the centre of the dipole, obtained from 2D PIC simulations with $a_0 = 0.6$, $\tau = 20$ fs, $\sigma = 40$ $\mu$m, $\lambda_{1,2} = 0.8$ $\mu$m and 0.779 $\mu$m, respectively, and $n_0 = 1.24 \times 10^{18}$ cm$^{-3}$ (10 THz). (a,c) $\gamma v_x/c$ vs. $x$ and (b,d) $\gamma v_y/c$ vs. $x$ of the electrons during (upper raw) and after (lower raw) the overlap of the driving laser pulses. The dipole centre is located at $x = 400$ $\mu$m, and the figures represent the electrons within 1 $\mu$m from the centre. The length of the dipole is comparable to the pulse width, i.e. of order $\sim 10$ $\mu$m.

## IV. OPTIMIZATION OF THE CONVERSION EFFICIENCY

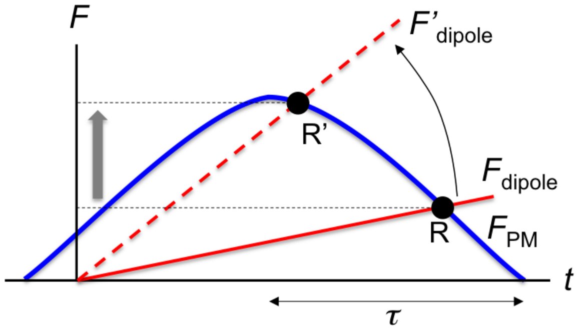


FIG. 8s: Ponderomotive force ($F_{\rm PM}$) and the electric restoring force ($F_{\rm dipole}$) due to the dipole field. The dipole field becomes the maximum for a given intensity of the laser pulse, when $F_{\rm dipole}$ meets $F_{\rm PM}$ at its peak, i.e. $R'$ in the figure. This can be achieved by increasing the slope of the growth of the dipole field, which is determined by the detuning of the laser pulses. Note that $R$ ($R'$) is the release point of the dipole block.

When we consider the PDO as a radiation source, the efficiency of the energy conversion from the driving laser pulses to the emitted radiation is one of the important parameters to determine the characteristics of the system. The theoretical maximum of the conversion efficiency can be estimated as follows. First, it is assumed that the release point of the dipole is made coincide with the peak of the driving ponderomotive force ($R'$ in Fig. 8s). In this way, the dipole field can be maximized for the given intensity of the laser pulses. From this condition, the slope of the dipole-growth is determined by the amplitude and duration of the driving laser pulses. At the release point, the electric force exerting on an electron by the dipole field ($E_{\rm di}$) balances the ponderomotive force, leading to

$$\begin{aligned} eE_{\rm di} = e\alpha S\tau =& \alpha\frac{e^2 n_0}{\epsilon_0} c\beta_\phi\tau = \alpha\eta mc\omega\frac{a_0^2}{\sqrt{1+a_0^2/2}} \simeq \alpha\eta mc\omega a_0^2, \\ \rightsquigarrow \quad & \bar{E}_{\rm di} = \alpha\frac{\omega_p^2}{\omega}\beta_\phi\tau \simeq \alpha\eta a_0^2, \end{aligned} \qquad \text{(s-13)}$$

where $\beta_\phi = \frac{v_\phi}{c} = \frac{\Delta\omega}{2ck}$ (i.e. the normalized velocity of the ponderomotive potential train), $\bar{E}_{\rm di} = eE_{\rm di}/(mc\omega)$, $\alpha$ the fraction of the trapped electrons and $\eta$ is the ensemble-average factor used in Eq. (4) of the article. Here we consider the cases, where $a_0 \leq 1$. The energy of the driving pulse per unit transverse area is $a_0^2 c\tau$, while the dipole field energy per unit

transverse area is $\bar{E}_{\rm di}^2 c\tau$. With $\zeta$, i.e. the conversion rate of $U_{\rm di}$ to the radiation energy $U_{\rm rad}$, the energy conversion efficiency is then, using (s-13),

$$\epsilon = \frac{\zeta U_{\rm di}}{U_{\rm laser}} = \zeta \frac{\bar{E}_{\rm di}^2 c\tau}{a_0^2 c\tau} = \zeta \alpha^2 \eta^2 a_0^2. \tag{s-14}$$

Eliminating $a_0^2$ using (s-13) yields

$$\epsilon = \zeta \alpha \eta \frac{\omega_p^2}{\omega} \beta_\phi \tau. \tag{s-15}$$

When the detuning is not that large (i.e. low $\beta_\phi$), the fraction of trapping is very close to unity ($\alpha \simeq 1$). The conversion rate of $U_{\rm di}$ to $U_{\rm rad}$ is roughly $\zeta \simeq 0.5$. The ensemble average factor is roughly $\eta \simeq 0.3$ (see the main article). With the additional phenomenological reduction factor $R \geq 0.6$ (see the main article), Eq. (s-15) becomes

$$\epsilon \simeq 0.1 \frac{\omega_p^2}{\omega} \beta_\phi \tau \tag{s-16}$$

Equation (s-15) indicates that the conversion efficiency can be increased considerably just by increasing the velocity of the ponderomotive potential train ($\beta_\phi$). In Fig. 6b and 6d of the main article, we examined two different detunings for the 20 THz case: $(\lambda_1, \lambda_2)$=$(1, 0.95)$ [$\mu$m] and $(1, 0.82)$ [$\mu$m]. For those cases, $\beta_\phi = |\lambda_1 - \lambda_2|/(\lambda_1 + \lambda_2)$ increases from 0.0256 to 0.0989. According to Eq. (s-15), the increase of the efficiency should be approximately by 4 times, while we obtained the increase by a factor of 5, which is reasonably close to the theoretical estimation.

Equation (s-15) or (s-16) indicates that the efficiency can be made very high by using a large $\beta_\phi$. However, practically, it is not easy for $\beta_\phi$ to be larger than 0.1. With $\omega \simeq 2\pi \times 300$ THz ($\lambda = 1$ $\mu$m), $\beta_\phi = 0.05$ and typical 30-fs driving pulses ($\tau \simeq 30$ fs), the efficiency to generate 20 THz radiation becomes $\epsilon \sim 10^{-3}$.

To obtain even higher efficiency, the pulse duration $\tau$ should be increased. However, it is not practically easy to use a very long pulse in exactly-head-on collisions of the laser pulses. As the dipole length is comparable to the pulse width, too large dipole length generated by very long pulses may result in breaking of the coherent block-like motion of the electrons. This problem can be remedied by colliding laser pulses obliquely as in Fig. 1. In this case, the dipole length can be maintained small enough even for very long pulses, by control of the collision angle and the pulse spot.

Potential problems that can arise in using long laser pulses are various instabilities originating from long interaction between the laser and plasma, and also the ion motion, which

is neglected in our analysis of the PDO. The laser-plasma instabilities can be made not to disturb the formation of the dipole easily by making the laser path inside the plasma as short as possible by control of the launching angle of the laser pulses and the plasma profile. Hence, the time scale of the ion motion may be the only limiting factor in increasing $\tau$. If we assume a single-ionized argon plasma, the ratio of ion and electron plasma frequencies, $\omega_{pe}/\omega_{pi} = 270$. This means that the ions remain stationary during a few hundreds of cycles of the electron oscillation. For 20 THz case, this amounts to tens of pico-seconds. Conservatively assuming much shorter pulse duration than that, for example, $\tau = 3000$ fs, the conversion efficiency can reach

$$\epsilon \sim 10^{-1}, \tag{s-17}$$

which is unprecedentedly high in milliJoule-order, tens-of-THz regime.
