# Supplementary figures and images for: High-Energy, Short-Duration Bursts of Coherent Terahertz Radiation from an Embedded Plasma Dipole
Source: Sci Rep. 2018 Jan 9;8:145. doi: 10.1038/s41598-017-18399-3 (PMC5760715; doi:10.1038/s41598-017-18399-3)

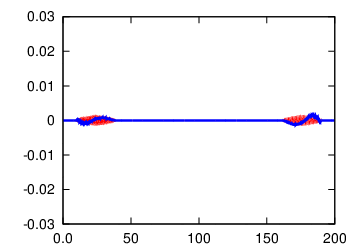

Supplement: Supplementary file 2 — Simulation of 1D dipole generation [file 41598_2017_18399_MOESM2_ESM.gif]
